# Predicting prolonged mechanical ventilation after endovascular treatment for acute vertebrobasilar artery occlusion: AIRFLOW score

**DOI:** 10.3389/fneur.2025.1673616

**Published:** 2025-11-14

**Authors:** Qiankun Cai, Minying Hong, Yingjie Xu, Shuai Zhang, Zhixin Huang, Pengfei Xu, Chunnuan Chen, Jixing Chen, Lichao Ye, Wen Sun

**Affiliations:** 1 Department of General Practice, The Second Affiliated Hospital of Fujian Medical University, Quanzhou, Fujian Province, China; 2 Department of Neurology, The First Affiliated Hospital of University of Science and Technology of China, Hefei, China; 3 Department of Neurology, Affiliated Hospital of Yangzhou University, Yangzhou, China; 4 Department of Neurology, Guangdong Second Provincial General Hospital, Guangzhou, China

**Keywords:** mechanical ventilation, vertebrobasilar artery, endovascular treatment, predictors, stroke

## Abstract

**Background and purpose:**

Vertebrobasilar artery occlusion (VBAO) is a rare yet severe type of ischemic stroke, often leading to respiratory failure that necessitates invasive mechanical ventilation and is associated with high mortality rates. While endovascular treatment (EVT) has improved outcomes for VBAO, many patients still require prolonged mechanical ventilation (PMV) post-EVT, further increasing mortality and posing challenging decisions for families. Currently, no predictive model exists to identify VBAO patients at risk of needing PMV after EVT. This study aims to develop and validate a predictive score for PMV in this patient population following EVT.

**Materials and methods:**

The derivation cohort prospectively recruited VBAO patients undergoing EVT from four comprehensive stroke centers (CSCs) in China. PMV was defined as continuous mechanical ventilation lasting for ≥7 days. Multivariable logistic regression was conducted to develop a scoring system. The performance of the model was evaluated for discrimination, calibration, and clinical utility. Four hundred and fourteen patients from acute Posterior circulation ischemic Stroke registry were enrolled to externally validate the model. Sensitivity analysis redefined PMV as using mechanical ventilation last for ≥ 14 days to further validate the model.

**Results:**

The derivation cohort consisted of 419 patients from four CSCs, among whom 113 (27.0%) required PMV. The presence of malignant cerebellar edema, posterior circulation collateral status, symptomatic intracranial hemorrhage post-EVT, atrial fibrillation, intravenous thrombolsis, vasopressor therapy and Glasgow coma score classification are found to be independent predictors of PMV in logistic regression, then ‘AIRFLOW’ scoring system was created. The AIRFLOW score demonstrated good discrimination in derivation cohort (C-index, 0.85, 95% CI 0.81 to 0.89), as well as the validation cohort (C-index, 0.82, 95% CI 0.77 to 0.86). Calibration plots and decision curve analysis for AIRFLOW score indicated that the model accurately predicted the risk of PMV and had satisfactory net benefit across various thresholds. Similar results were found in sensitivity analysis.

**Conclusion:**

The AIRFLOW score may help predict PMV in VBAO patients after EVT.

## Introduction

Vertebrobasilar artery occlusion (VBAO) is a rare but severe form of ischemic stroke, accounting for 1% of all strokes and 5% of large vessel occlusions (LVOs) (1, 2). Unlike anterior circulation LVOs, VBAO frequently necessitates invasive mechanical ventilation because it injures brainstem centers that regulate consciousness, central respiratory drive, and airway protective reflexes; posterior fossa complications (e.g., malignant cerebellar edema, progressive brainstem infarction) further predispose patients to central hypoventilation, severe neurogenic dysphagia, and aspiration pneumonia, thereby prolonging ventilation and contributing to higher disability and mortality (3, 4). Recent meta-analysis has demonstrated that combining endovascular treatment (EVT) with optimal medical care improves functional outcomes at 90 days compared to medical care alone (5). In a comprehensive, nationally representative cohort of hospitalized acute ischemic stroke patients in the US, approximately 16.7% of those undergoing EVT needed prolonged mechanical ventilation (PMV). This group had a significantly higher in-hospital mortality rate compared to those who did not require PMV (32.1% vs. 8.2%). It’s important to note that the study primarily focused on patients with anterior circulation LVOs (6). Additionally, when a patient require PMV, family members not only face high medical expenses but also the tough decision of whether to continue treatment in hopes of a good outcome.

Given the distinct divergence in MV requirements and outcomes between anterior and posterior circulation LVOs patients, coupled with the encouraging advancements in EVT for VBAO, there is a clear lack of a predictive model for identifying VBAO patients at risk of requiring PMV after EVT. Our study aims to develop and validate a risk model to predict PMV post-EVT for VBAO patients. We presented this article in accordance with TRIPOD Checklist.

## Materials and methods

### Derivation cohort

We enrolled VBAO patients who underwent EVT at four comprehensive stroke centers (First Affiliated Hospital of University of Science and Technology of China [USTC], Guangdong second Provincial General Hospital, the Second Affiliated Hospital of Fujian Medical University [FJMU], The Affiliated hospital of Yangzhou University [YZU]) between January 2019 and June 2022. Inclusion criteria were: (1) age ≥18 years; (2) imaging confirmation of acute symptomatic VBAO; and (3) EVT performed within 24 h of estimated occlusion. Patients with only posterior cerebral artery occlusion treated with EVT and a pre-stroke modified Rankin Scale (mRS) score >2 were excluded. Additionally, we excluded patients without MV information (*n* = 12), those who withdrew life-sustaining treatment (WLST) within 7 days after hospitalization (*n* = 74), as well as those who died within the first 7 days while on MV (*n* = 64).

### External validation cohort

The acute Posterior circulation ischemic Stroke registry (PERSIST) database is a retrospective registry of patients with acute VBAO who underwent EVT within 24 h of estimated occlusion time (EOT) between December 2015 and December 2018 at 21 stroke centers in 13 provinces of China (http://www.chictr.org.cn/; unique identifier: ChiCTR2000033211). Details of the PERSIST study have been described previously (7–9). In this study, we excluded patients lacking MV data (*n* = 58), those who died within the first 7 days of initiating MV (*n* = 52), and patients with WLST during the first 7 days after hospitalization (*n* = 65).

### Weaning protocol

Generally, all patients were monitored in neurocritical intensive care unit for at least 24 h after EVT. Patients on MV were evaluated daily by trained respiratory therapists and physicians at each center with the aim of weaning them as soon as possible according to the predefined weaning criteria: (1) stable cardiovascular status (heart rate[HR] ≤ 140 beats/min, systolic blood pressure[SBP] 90–160 mmHg, and minimal or absence of catecholamine); (2) adequate oxygenation (oxygen saturation measured by pulse oximetry [Spo_2_] ≥ 90%, fractional inspired oxygen tension [Fio_2_] ≤ 40%, positive end-expiratory pressure ≤8 cm H_2_O, respiratory rate ≤35 breaths/min); (3) PaCO_2_ ≤ 50 mmHg; (4) temperature <38.5 °C; (5) pH > 7.35. When patients met the criteria above, a spontaneous breathing trial was performed with 30-min T-tube trial or ventilatory support level ≤ 7 to 8 cm H_2_O. Weaning test failure was defined by the development of any the following criteria within 30 min: respiratory rate >35 breaths/min with increased accessory muscle activity, Spo_2_ < 90% (on Fio_2_ = 0.4), HR > 140 beats/min, SBP < 90 mmHg or >180 mmHg, major dyspnea or agitation (10). The determination of weaning failure is to be made by trained respiratory therapists and physicians at each center.

### Data collection

The data on patient characteristics, medical history, level of fasting blood glucose (FBG), stroke cause, pre-treatment imaging parameters, treatment-related factors, and clinical examination and radiographic results at follow-up were meticulously gathered. All patients underwent regular clinical examinations by experienced physicians at each center. The severity of strokes was evaluated using the National Institutes of Health Stroke Scale (NIHSS) score at admission. Consciousness was assessed with the Glasgow Coma Scale (GCS) score on admission, categorizing patients into a severe consciousness impairment group (3–8 points) and a moderate consciousness impairment group (9–15 points).

Imaging parameters were collected, including infarction core status, assessed using the posterior circulation Alberta Stroke Program Early CT Score (pc-ASPECTS) on CT or MRI (11); Posterior circulation collateral status evaluated through the Basilar artery on CT angiography (BATMAN) was categorized into two groups: less than 7 points indicating poor collateral and 7–10 points indicating good collateral (12); and reperfusion status determined according to modified Thrombolysis in Cerebral Infarction scale on final angiography, with scores of 2b or 3 indicating successful reperfusion (13). The occlusion site was categorized as either ‘basilar artery’ or ‘basilar artery plus vertebral artery’. The basilar artery plus vertebral artery occlusion was characterized as an occlusion of vertebral artery resulting in no flow to basilar artery (9).

Typically, patients underwent CT or MRI scans within 72 h post-EVT for radiographic assessment. Symptomatic intracranial hemorrhage (sICH) was defined as neuroimaging-identified intracranial hemorrhage coupled with ≥4-point increase in NIHSS score or an increase of >2 point for each item (14). Malignant cerebellar edema (MCE) was determined using the Jauss scale for neuroradiological mass effect, with a score of ≥4 indicating MCE (15, 16). All neuroimaging data were sent to the core laboratory of USTC and evaluated blindly by two experienced neuroradiologists. For instances of disagreement, decisions were made by a third experienced neuroradiologist.

Treatment-related factors such as the choice of first-line treatment, intravenous thrombolysis (IVT), time from EOT to puncture, time from puncture to reperfusion, type of anesthesia administered, and vasopressor therapy (defined as the use of vasoconstrictors like norepinephrine, epinephrine, aramine, and dopamine to manage shock caused by various underlying conditions during hospitalization) (17) were also documented.

### Outcomes

Following a previous study (18), the development of PMV was defined as continuous invasive MV lasting for 7 days or more. Data was collected from anonymized electronic medical record system and hospital information system by two experienced physicians (S. Z and L. Y).

### Statistical analysis

The data were presented as the median with interquartile range or numbers with percentages. Univariate analysis was performed using the *χ*^2^ test for dichotomous variables and Mann–Whitney *U* test for continuous variables to compare patients with and without PMV in derivation cohort. For the missing variables, multivariate imputation by chained equation was performed using the fully conditional specification approach.

To identify the independent predictors of PMV, we implemented a multivariable logistic regression analysis (forward stepwise method based on maximum likelihood estimation) including variables with a *p*-value <0.05 in univariate analysis of derivation cohort. Regression coefficients, odds ratios (OR), and 95% confidence intervals (95% CI) for each variable in the model were calculated. Based on the regression coefficients from the model, we calculated the corresponding points on the scale. We created a scoring system by rounding to the nearest whole number and assigning integer values (19).

To evaluate prediction accuracy, we used three methods: concordance index (C-index) with 95% CI to measure discrimination, calibration plot to measure model fit, and decision curve analysis (DCA) to measure clinical utility. Internal validation was performed by generating 1,000 bootstrap resamples from the derivation cohort to ensure robustness. External validation was conducted using data from the validation cohort. A clinically useful C-index was considered to be ≥ 0.70.

In sensitivity analysis, we redefined PMV as continuous invasive MV lasting for 14 days or more. Thus, we excluded patients who died within 14 days while on MV and those who had WLST within 14 days after hospitalization in both cohorts. The scoring system was then validated using the remaining patients from both cohorts.

The performance of the new scoring system was then compared with the previously published risk model (SET score, Supplementary Table S1) (20) using the DeLong method. All statistical analyses were performed with SAS version 9.4 and R version 4.3.1. A two-sided *p*-value <0.05 was considered statistically significant.

## Results

**Figure 1:**
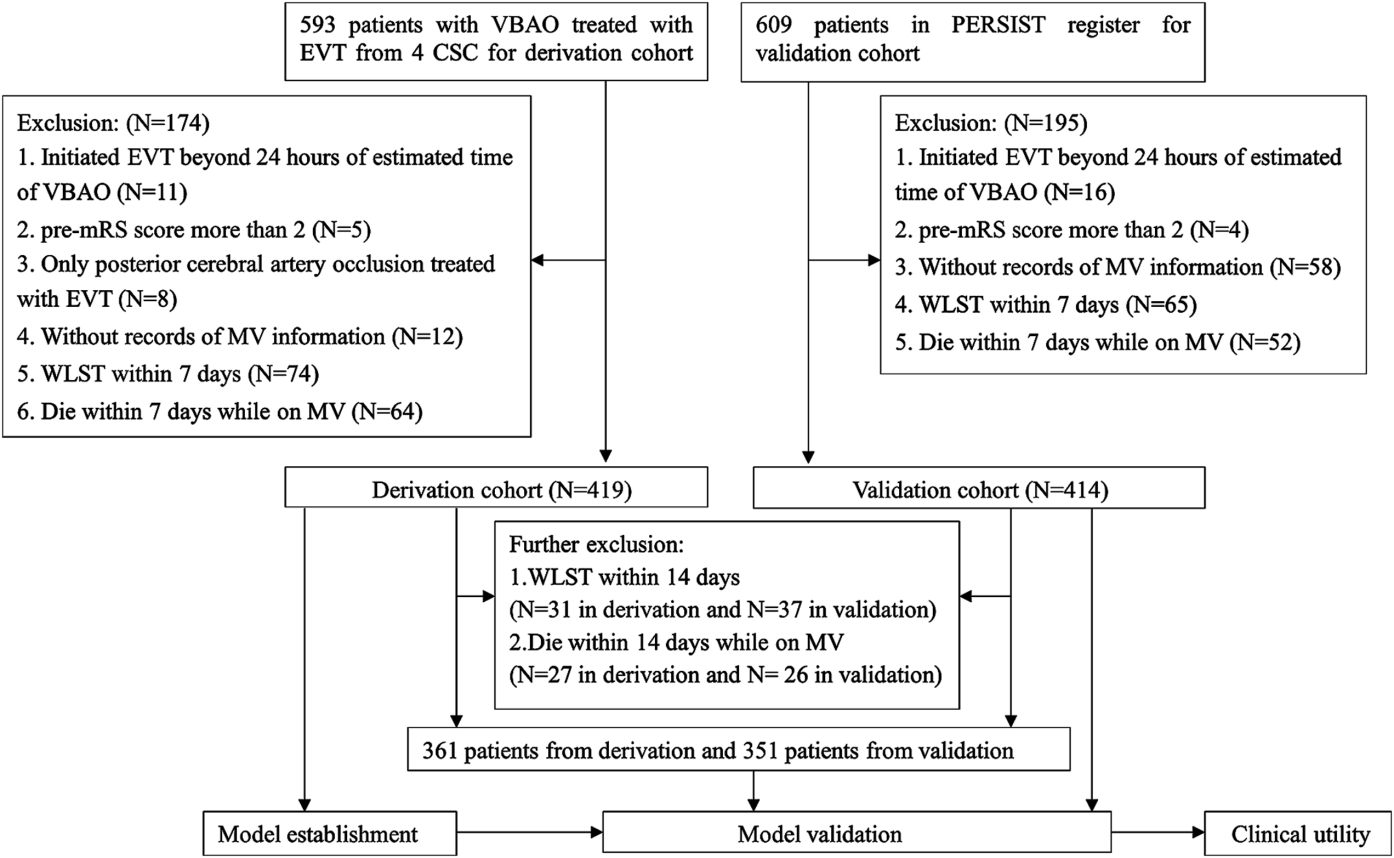
Flow chart of patient inclusion. VBAO, vertebrobasilar artery occlusion; EVT, endovascular treatment; CSC, comprehensive stroke centers; PERSIST, acute posterior circulation ischemic stroke registry; mRS, modified Rankin Scale; MV, mechanical ventilation; WLST, withdraw life-sustaining treatment.

The study included 593 VBAO patients who underwent EVT in the derivation cohort and 609 patients in the validation cohort. Of these, 419 patients (median age 64 years, 27.4% female) from the derivation cohort and 414 patients (median age 66 years, 25.8% female) from the validation cohort were eligible for analysis. Figure 1 presents the flowchart for patient selection, and Table 1 shows the characteristics of patients in both cohorts.

**Table 1:** Baseline characteristics of the two cohorts.

	Derivation cohort	Validation cohort	*P*
	*N* = 419	*N* = 414	
Age, years	64(55–73)	66(56–73)	0.463
Sex			0.601
Male	304(72.6)	307(74.2)	
Female	115(27.4)	107(25.8)	
Medical history			
Hypertension	279(66.6)	288(69.6)	0.357
Diabetes mellitus	93(22.2)	100(24.2)	0.503
Hyperlipidemia	159(37.9)	143(34.5)	0.307
Atrial fibrillation	96(22.9)	85(20.5)	0.405
Previous stroke	81(19.3)	94(22.7)	0.232
Coronary artery disease	40(9.5)	42(10.1)	0.702
Current smoking	136(32.5)	124(30.0)	0.435
Drink	90(21.5)	87(21.0)	0.870
Fasting blood glucose, mmol/L	7.3(6.0–9.6)	6.7(5.8–8.4)	**<0.001**
Clinical examination			
NIHSS	22(13–28)	22(10–28)	0.348
GCS classification			**<0.001**
Moderate impairment	197(47.0)	252(60.9)	
Severe impairment	222(53.0)	162(39.1)	
Stroke cause			0.560
Large artery atherosclerosis	273(65.2)	255(61.6)	
Cardioembolism	88(21.0)	95(22.9)	
Other	21(5.0)	18(4.3)	
Undetermined	37(8.8)	46(11.1)	
Pre-treatment imaging parameters			
pc-ASPECTS	9(8–10)	8(7–10)	**<0.001**
Collateral circulation status			**<0.001**
Good	93(22.2)	160(38.6)	
Poor	326(77.8)	254(61.4)	
Site of occlusion			0.791
Basilar artery	295(70.4)	288(69.6)	
Basilar artery + vertebral artery	124(29.6)	126(30.4)	
Treatment-related factors			
Intravenous thrombolysis	87(20.8)	113(27.3)	0.027
Estimated occlusion to puncture time, minutes	300(215–455)	210(90–366)	**<0.001**
Puncture to reperfusion time, minutes	105(69–148)	110(75–150)	0.483
Type of anesthesia			**<0.001**
General anesthesia	101(24.2)	191(46.1)	
Sedative	317(75.8)	223(53.9)	
First-line treatment			**<0.001**
Stent retriever	272(64.9)	344(83.1)	
Aspiration	83(19.8)	25(6.0)	
Balloon angioplasty or stenting	54(12.9)	41(9.9)	
Intra-arterial thrombolysis	10(2.4)	4(1.0)	
Rescue therapy	196(46.8)	120(29.3)	**<0.001**
Successful reperfusion	377(90.0)	381(92.0)	0.301
Vasopressor therapy	80(19.1)	48(11.6)	**0.003**
Radiographic results at follow-up			
sICH	26(6.2)	23(5.6)	0.690
Malignant cerebellar edema	56(13.4)	49(11.8)	0.506

Data are present as *n* (%). Continuous variables are expressed as median (interquartile range). GCS, Glasgow coma scale; NIHSS, National Institutes of Health Stroke Scale; pc-ASPECTS, posterior circulation-Alberta Stroke Program Early CT score; sICH, symptomatic intracranial hemorrhage. The bold number indicate statistical significance.

In the derivation cohort, 113 patients (27.0%) required PMV for a median of 12 days (IQR, 8.5–16 days). In the validation cohort, 102 (24.6%) needed PMV for a median of 10 days (IQR, 9–16 days). The distribution of mRS scores at 90 days based on PMV status (Supplementary Figure S1) and the survival probability of patients with PMV in the two cohorts are shown in Supplementary Figure S2.

### Model development

A comparison of baseline characteristics, medical history, clinical examination, treatment-related factors, and imaging parameters between non-PMV and PMV in derivation cohort is shown in Table 2. Compared with non-PMV patients, PMV patients were significantly less likely to be female (*p* = 0.048), have atrial fibrillation (AF) (*p* = 0.039), receive IVT (*p* < 0.001), and have good collateral (*p* < 0.001). They were more likely to have severe consciousness impairment and higher NIHSS score (both *p* < 0.001) and higher FBG level (*p* = 0.007). They also had a higher proportion of vasopressor therapy, presence of sICH, and MCE at follow-up imaging (all *p* < 0.001).

**Table 2:** Results of the univariable and multivariable logistic regression for PMV after EVT in the derivation cohort.

	Univariable			Multivariable logistic		
	PMV	Non-PMV	*P*	β	OR (95% CI)	*P*
	*n* = 113	*n* = 306				
Age, years	65(55–73)	64(55–73)	0.717			
Sex			**0.048**			
Male	90(79.6)	214(69.9)				
Female	23(20.4)	92(30.1)				
Hypertension	80(70.8)	199(65.0)	0.267			
Diabetes mellitus	25(22.1)	68(22.2)	0.983			
Hyperlipidemia	43(38.1)	116(37.9)	0.978			
Atrial fibrillation	18(15.9)	78(25.5)	**0.039**	−0.88	0.42(0.21–0.84)	**0.014**
Previous stroke	21(18.6)	60(19.6)	0.814			
Coronary artery disease	13(11.5)	27(8.8)	0.407			
Current smoking	32(28.3)	104(34.0)	0.271			
Drink	20(17.7)	70(22.9)	0.252			
Fasting blood glucose, mmol/L	8.0(6.3–11.0)	7.3(5.9–9.1)	**0.007**			
National Institutes of Health Stroke Scale	27(19–31)	19(11–28)	**<0.001**			
Glasgow coma score classification			**<0.001**			**0.009**
Moderate impairment	30(26.5)	167(54.6)		-	reference	**–**
Severe impairment	83(73.5)	139(45.4)		0.74	2.10(1.21–3.66)	
Stroke cause			0.682			
Large artery atherosclerosis	72(63.7)	201(65.7)				
Cardioembolism	22(19.5)	66(21.6)				
Other	6(5.3)	15(4.9)				
Undetermined	13(11.5)	24(7.8)				
pc-ASPECTS	9(7–10)	9(8–10)	0.567			
Collateral circulation status			**<0.001**			**0.001**
Good	6(5.3)	87(28.4)		–	reference	
Poor	107(94.7)	219(71.6)		1.61	5.03(1.99–12.78)	
Site of occlusion			0.537			
Basilar artery	77(68.1)	218(71.2)				
Basilar artery + vertebral artery	36(31.9)	88(28.8)				
Intravenous thrombolysis	9(8.0)	78(25.5)	**<0.001**	−1.26	0.29(0.12–0.67)	**0.004**
Estimated occlusion to puncture time, minutes	270(199–444)	310(222–455)	0.373			
Puncture to reperfusion time, minutes	105(70–145)	105(67–150)	0.710			
Type of anesthesia			0.310			
General anesthesia	31(27.7)	70(22.9)				
Sedative	81(72.3)	236(77.1)				
First-line treatment			0.832			
Stent retriever	77(68.1)	195(63.7)				
Aspiration	20(17.7)	63(20.6)				
Angioplasty or stenting	14(12.4)	40(13.1)				
Intra-artery thrombolysis	2(1.8)	8(2.6)				
Rescue therapy	59(52.2)	137(44.8)	0.175			
Successful reperfusion	97(85.8)	280(91.5)	0.087			
Vasopressor therapy	47(41.6)	33(10.8)	**<0.001**	1.59	4.90 (2.68–8.96)	**<0.001**
Symptomatic intracranial hemorrhage	16(14.2)	10(3.3)	**<0.001**	1.19	3.27(1.24–8.67)	**0.017**
Malignant cerebellar edema	34(30.1)	22(7.2)	**<0.001**	1.50	4.48(2.19–9.17)	**<0.001**

Data are present as *n* (%). Continuous variables are expressed as median (interquartile range). EVT, endovascular treatment; PMV, prolonged mechanical ventilator; pc-ASPECTS, posterior circulation-Alberta Stroke Program Early CT score. The bold number indicate statistical significance.

Multivariate logistic analysis found that malignant cerebellar edem***a*** at follow-up imaging, posterior c***i***rculation collateral status, symptomatic intracranial hemo***r***rhage post-EVT, atrial ***f***ibrillation, intravenous thrombo***l***ysis, vas***o***pressor therapy and Glasgo***w*** coma score classification was independently associated with PMV (Table 2). The ‘AIRFLOW’ scores were then developed based on the *β* coefficients of individual variables (Table 3).

**Table 3:** Components of the AIRFLOW grading scale.

Items	Categories	Points
Malignant cerebellar edem***a***	Yes	2
	No	0
posterior collateral c***i***rculation status	Poor	2
	Good	0
Symptomatic intracranial hemo***r***rhage	Yes	2
	No	0
Atrial ***f***ibrillation	No	1
	Yes	0
Intravenous thrombo***l***ysis	No	2
	Yes	0
Vas***o***pressor therapy	Yes	2
	No	0
Glasgo***w*** coma score classification	Severe impairment	1
	Moderate impairment	0
Total scores:		

The C-index for the AIRFLOW score was 0.85 (95% CI 0.81 to 0.89) in derivation cohort. The internal validation yielded a bootstrap-corrected C-index of 0.85 (95% CI 0.81 to 0.89). Calibration plots of the AIRFLOW score in both the derivation and internal validation (Figure 2A) demonstrated that the predicted probabilities of PMV were close to the observed PMV.

**Figure 2:**
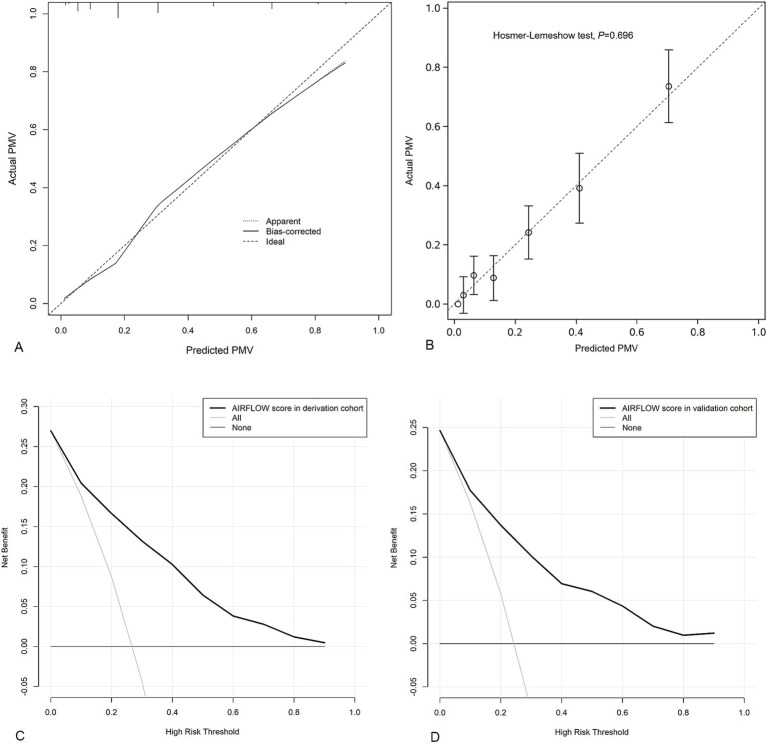
Calibration plots to assess model internal validation with 1,000 bootstrap resamples; The dotted line represents the performance of the AIRFLOW score. The solid line corrects for any bias in the AIRFLOW score. The dashed line is the reference line where an ideal AIRFLOW score would lie **(A)**. Calibration plots of external validation for the AIRFLOW score **(B)**. Decision curve analysis demonstrating the net benefit associated with the use of the AIRFLOW score for predicting PMV in derivation cohort **(C)** and validation cohort **(D)**. PMV prolonged mechanical ventilation.

### Model validation

In the external validation, the AIRFLOW score had a C-index of 0.82 (95% CI 0.77 to 0.86). Calibration plots of the AIRFLOW score indicated that the model accurately predicted the risk of PMV (Figure 2B).

### Clinical utility

Based on DCA in derivation cohort the AIRFLOW scores to predict PMV provided more net benefit than either a “treat-all-patients scheme” or a “treat-none scheme” (Figure 2C). These findings were consistent in the validation cohort as well (Figure 2D).

### Risk stratification

**Figure 3:**
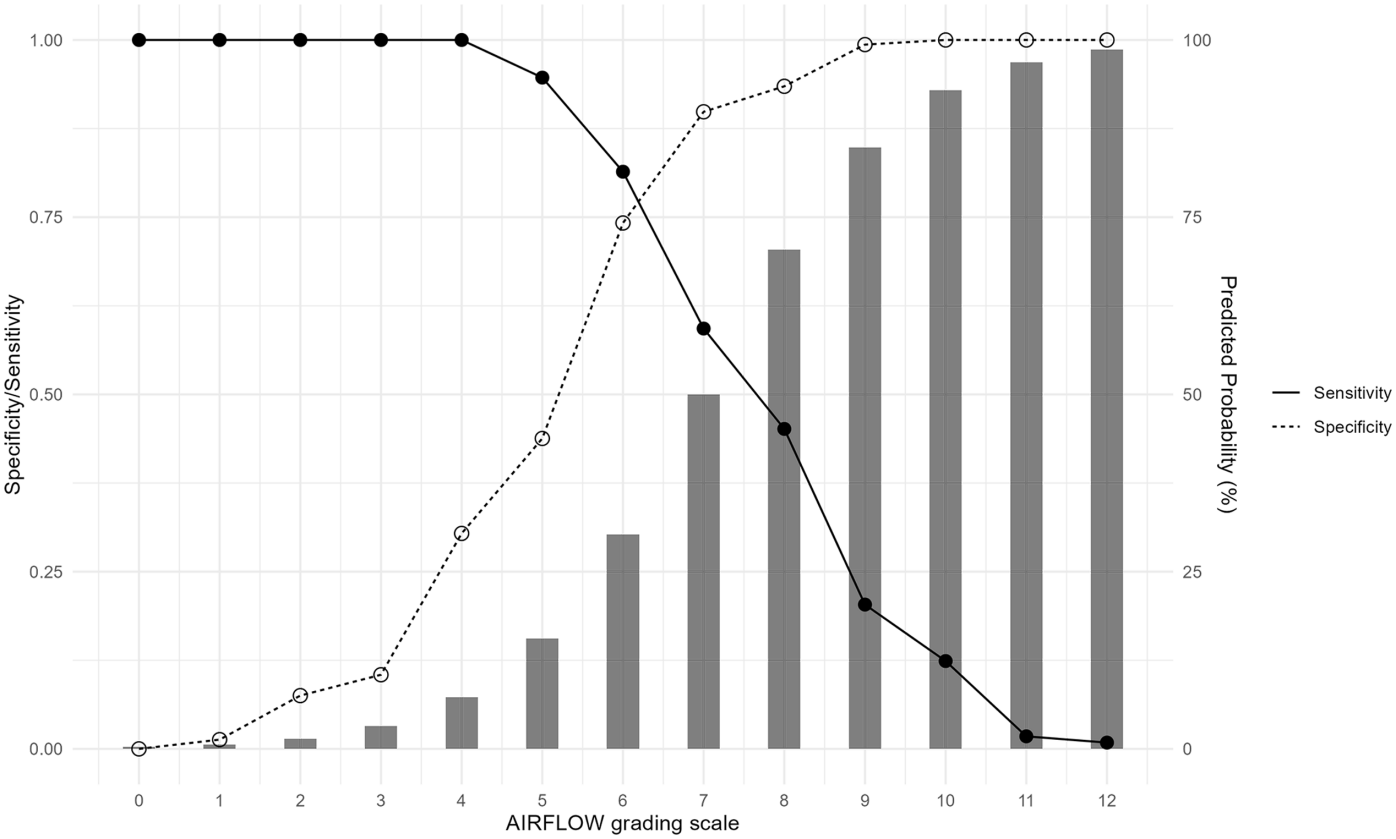
The change of sensitivity (solid line), specificity (dashed line) and predicted probability (bars) of requiring prolonged mechanical ventilation as AIRFLOW score increases.

In the derivation cohort, the maximum sum AIRFLOW score was 12 points. A patient with a score of 0 has P_PMV_ = 0.3%, and a patient with a full score of 12 has P_PMV_ = 98.6%. As the sum AIRFLOW scores increased, the predicted probability of PMV also increased gradually (Figure 3 and Supplementary Table S2).

A sum AIRFLOW score of ≤5 points indicated a low risk (0–25%) for PMV, 6–7 points indicated an intermediate risk (26–50%), and ≥8 points indicated a high risk (>50%) for PMV. The association between three risk stratification of sum AIRFLOW score and actual PMV is displayed Supplementary Figure S3.

### Sensitivity analysis

When PMV was redefined as using continuous invasive MV last for ≥14 days, the AIRFLOW score was used to predict the risk of PMV. A C-index of 0.84 (95% CI 0.79–0.88) was obtained from remaining patients in derivation cohort and of 0.82 (95% CI 0.76–0.87) in validation cohort. Both calibration plots of the AIRFLOW score indicated that the model accurately predicted the risk of PMV (Supplementary Figures S4A,B). Moreover, based on DCA, the AIRFLOW score to predict PMV would provide more net benefit than either a “treat-all-patients scheme” or a “treat-none scheme” in both cohorts (Supplementary Figures S4C,D).

### Model comparison

In the derivation cohort with complete data on items of SET score, the AIRFLOW score demonstrated superior discriminative performance compare to the SET score, reflected by a higher C-index (0.85, 95%CI 0.81–0.89, vs. 0.67, 95%CI 0.61–0.74, *p* < 0.001). This superiority was corroborated in the external validation cohort with complete data on items of SET score, where the AIRFLOW score maintained a higher C-index (0.82, 95%CI 0.77–0.87, vs. 0.72, 95%CI 0.66–0.78, *p* < 0.001, Supplementary Figures S5A,B) compared to the SET score. Furthermore, sensitivity analysis with complete data on items of SET score also underscored the AIRFFLOW score’s enhanced performance over the SET score across both cohorts (Supplementary Figures S5C,D).

## Discussion

This study demonstrated that prolonged mechanical ventilation (PMV) is commonly needed in vertebrobasilar artery occlusion (VBAO) patients treated with endovascular treatment (EVT). Seven readily accessible variables were identified as potential predictors for PMV: the presence of malignant cerebellar edem**a** on follow-up imaging, posterior c**i**rculation collateral status, symptomatic intracranial hemo**r**rhage after EVT, atrial **f**ibrillation, intravenous thrombo**l**ysis, vas**o**pressor therapy and Glasgo**w** coma score classification. Utilizing these metrics, the ‘AIRFLOW’ scoring system was devised, showing strong discriminative capabilities and a good model fit. Its validity was confirmed through internal validation using 1,000 bootstrap resamples, along with external validation procedures.

In patients with VBAO undergoing EVT who require PMV, poor outcomes are prevalent. Specifically, our study reveals that less than 10% of patients achieve favorable outcomes, with more than 60% mortality within 90 days. It is crucial to highlight that, in addition to severe impairment in brainstem areas controlling consciousness, respiration, and circulation, which contribute to premature in-hospital mortality, the withdrawal of life-sustaining therapy (WLST) during hospitalization stands out as a pivotal determinant of patient outcomes. Prior research also indicates that severe central nervous system injury is a primary factor in WLST decisions in intensive care settings (21).

In our study, PMV was defined as mechanical ventilation for 7 days or more, deviating from the standard threshold of ≥21 days (22) to enhance inclusivity, considering the high rate of in hospital mortality or WLST before reaching 21 days. A supplementary sensitivity analysis was conducted, redefined PMV as mechanical ventilation lasting 14 days or more. Despite this adjustment, the AIRFLOW scoring system retained its predictive accuracy and reliability. On the contrary, according to the definition of PMV, we cannot include patients who die within 7 days of using MV in the analysis. In clinical practice, when a severe stroke requires MV, physicians and some family members often agree to a time-limited trial to see if the patient’s condition improves or deteriorates (23). Therefore, predicting the use of MV within 7 days might not be crucial.

### Mechanism of associations

The presence of MCE and sICH after EVT contributes to obstructive hydrocephalus and cerebellar tonsillar herniation. These conditions can cause secondary injury to the brainstem due to mass effect, increasing the risk of impaired consciousness and failure of respiratory or circulatory functions, both of which are radiographically linked to PMV (20).

Collateral circulation status, as evaluated by BATMAN score, plays a pivotal role in the pathophysiology of cerebral ischemia. An unfavorable BATMAN score (less than 7 points) significantly correlates with a poor outcome (12), likely due to accelerated progression of the ischemic penumbra into infarction. This leads to a large infarct affecting brainstem respiratory or circulatory centers.

Interestingly, a history of AF emerged as a protective factor against PMV in VBAO patients post-EVT. This may be because the cardioembolic mechanism often underlying VBAO in AF patients is linked to higher rates of successful recanalization compared to atherosclerotic mechanisms (24, 25). Consequently, this might reduce damage to critical brainstem centers.

The EVT with IVT first was associated with better functional outcomes in patients with VBAO treated within 24 h of onset (26). Potential advantages of bridging therapy include early thrombus fragmentation, microvascular reperfusion and enhanced recanalization (27, 28). In clinical practice, patients with acute ischemic stroke presenting within 4.5 h of symptom onset are prioritized for IVT. This suggests that patients receiving IVT present earlier medically than those who do not, resulting in shorter onset-to-treatment times and improved outcome (29). These effects help salvage significant amounts of cerebral tissue, potentially reducing damage to critical brainstem centers.

Vasopressor therapy emerged as the strong predictor for PMV in the AIRFLOW score. Patients requiring vasopressor therapy often struggle to meet the weaning criteria due to unstable cardiovascular status. Additionally, vasopressor therapy may signify impairment of the cardiovascular regulating center in the medulla, potentially caused by infarction or intracranial hypertension, thereby increasing the risk of cardiac arrest (1).

Finally, severe consciousness impairment (Glasgow coma score of 8 points or less), which reduces protection reflexes and increases the risk for aspiration, is associated with a high need for tracheostomy or a higher rate of extubation failure (20, 30).

### Clinical implications

To our best knowledge, the AIRFLOW scoring system is the first designed specifically to predict the risk of PMV in patients with VBAO treated with EVT. Compared to the SET score, the AIRFLOW score requires fewer items and offers superior discriminatory performance.

The AIRFLOW score serves as a clinical tool that helps stratify patients into distinct risk categories for PMV. It assists clinicians in making informed decisions, communicating effectively with patients’ relatives, and optimizing patient care. For patients with a low risk who are currently on MV, this scoring system predicts a good chance of eventually independent breathing. This provides peace of mind for patients and their families helps avoid premature decisions to WLST. For high-risk patients using MV, considering a time-limited trial is advisable while respecting their wishes and values. If needed, moving toward comfort care afterward is a logical next step. Especially when the AIRFLOW score exceeds 9, the specificity reaches 100% (false positive rate of zero).

Notably, the AIRFLOW scoring system includes a unique adjustable factor—the administration of IVT before EVT. This introduces a specific strategy for intervention in high-risk groups. Increasing the frequency of IVT may reduce the occurrence of PMV after EVT, thereby substantially improving patient outcomes.

Our study has several limitations. Firstly, we lacked complete data on the SET score items, many of which could be potential predictors for PMV. Missing key variables means the model might not capture all factors affecting the outcomes, reducing prediction accuracy. Secondly, we excluded patients on MV who died or had life-sustain treatment withdrawn within 7 days. This exclusion could lead to survivorship bias, making model overly optimistic by ignoring patients who died or ceased treatment quickly due to severe conditions. Thirdly, our dataset lacked the Full Outline of UnResponsiveness (FOUR) score. FOUR captures brainstem reflexes and respiratory patterns and may be more sensitive than GCS for posterior circulation stroke, but its required variables were not collected—especially in the validation cohort—and cannot be reconstructed retrospectively. We therefore used GCS, which was consistently available and clinically relevant to airway protection and ventilator weaning. This gap may underrepresent brainstem dysfunction; prospective studies should record both GCS and FOUR to enable direct comparison and potentially improve PMV prediction. Lastly, the high rate of catastrophic outcomes in patients need PMV may partly result from premature WLST decision. Further external validation of the AIRFLOW score and PMV outcomes is necessary.

## Conclusion

In this study, we identified seven variables linked to PMV in VABO patients that can be easily assessed after EVT. We propose the AIRFLOW score to estimate the likelihood of PMV. These findings can assist patient families and caregivers in better understand the treatment path. Further validation of the AIRFLOW score’s effectiveness is necessary.

## Data Availability

The raw data supporting the conclusions of this article will be made available by the authors, without undue reservation.
