# Supplementary material for: Predicting prolonged mechanical ventilation after endovascular treatment for acute vertebrobasilar artery occlusion: AIRFLOW score
Source: Front Neurol. 2025 Nov 14;16:1673616. doi: 10.3389/fneur.2025.1673616 (PMC12660248; doi:10.3389/fneur.2025.1673616)
Supplement: Supplementary file 1 [file Table_1.DOCX]

**Supplementary materials**

**Title:** Predicting prolonged mechanical ventilation after endovascular treatment for acute vertebrobasilar artery occlusion：AIRFLOW score

**
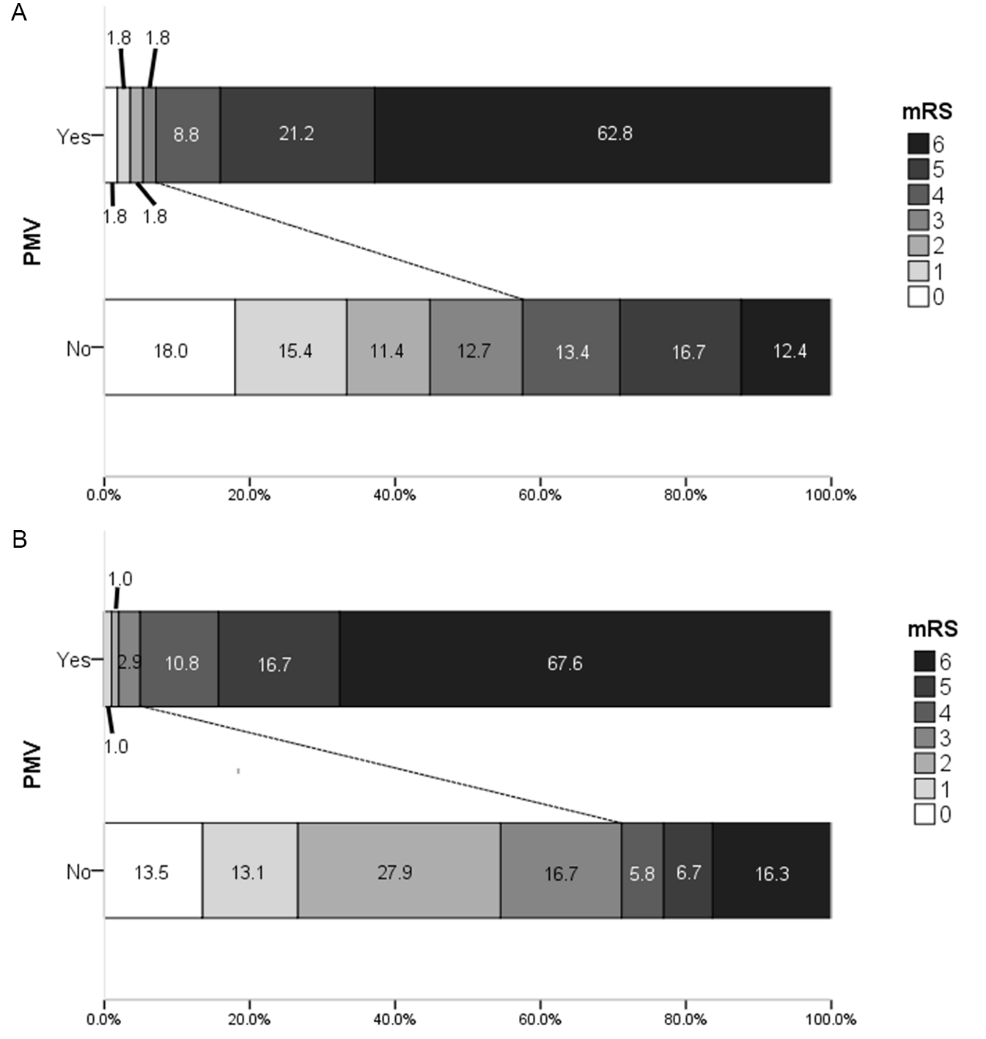
**

**Figure S1** Distribution of mRS at 90 days in vertebrobasilar artery occlusion patients treated with endovascular treatment according to PMV status in derivation cohort (A) and validation cohort (B). Unfavorable outcome was defined as mRS of 4-6. PMV prolonged mechanical ventilation; mRS modified Rankin Scale.


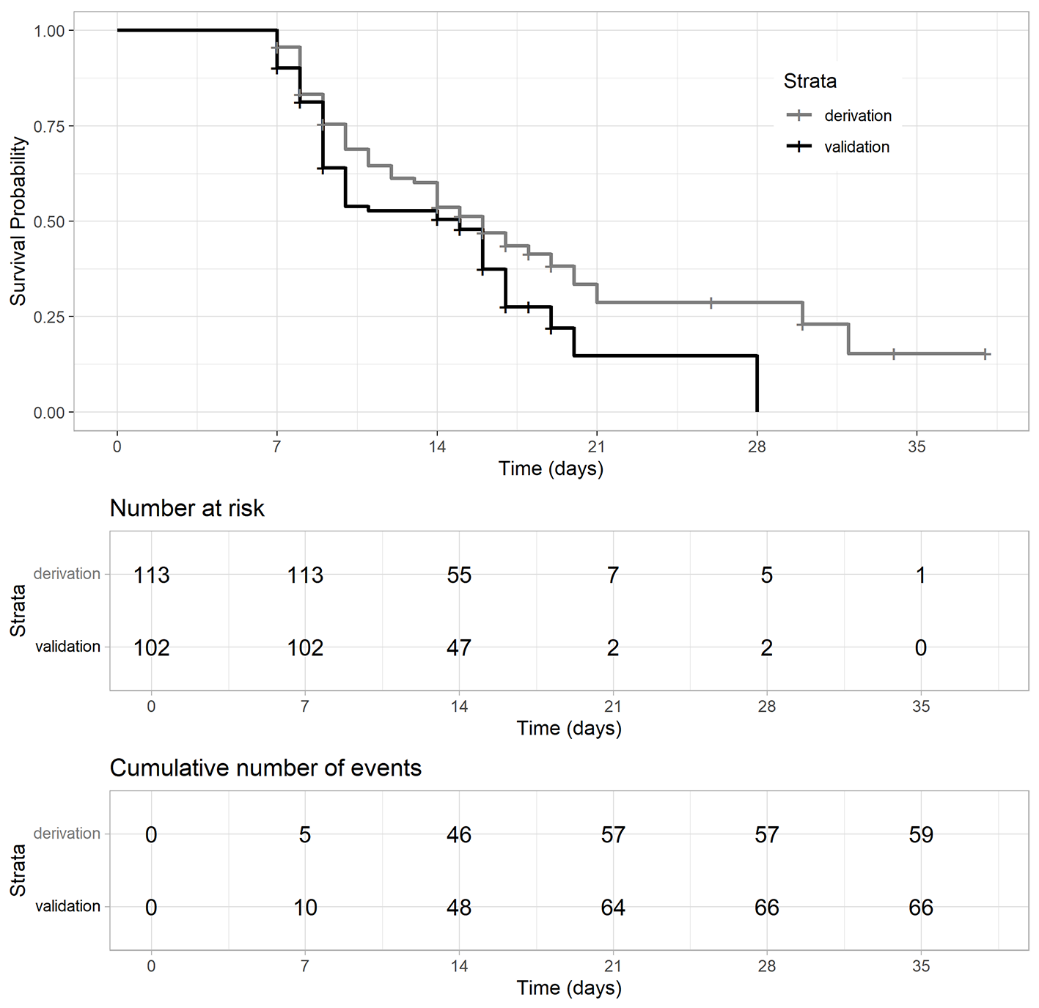


**Figure S2** The survival probability of patients with prolonged mechanical ventilation from both cohorts. The endpoint event was in-hospital mortality or withdrawn life-sustaining treatment during hospitalization. Censoring was done if the patient successfully weaned off the mechanical ventilation.
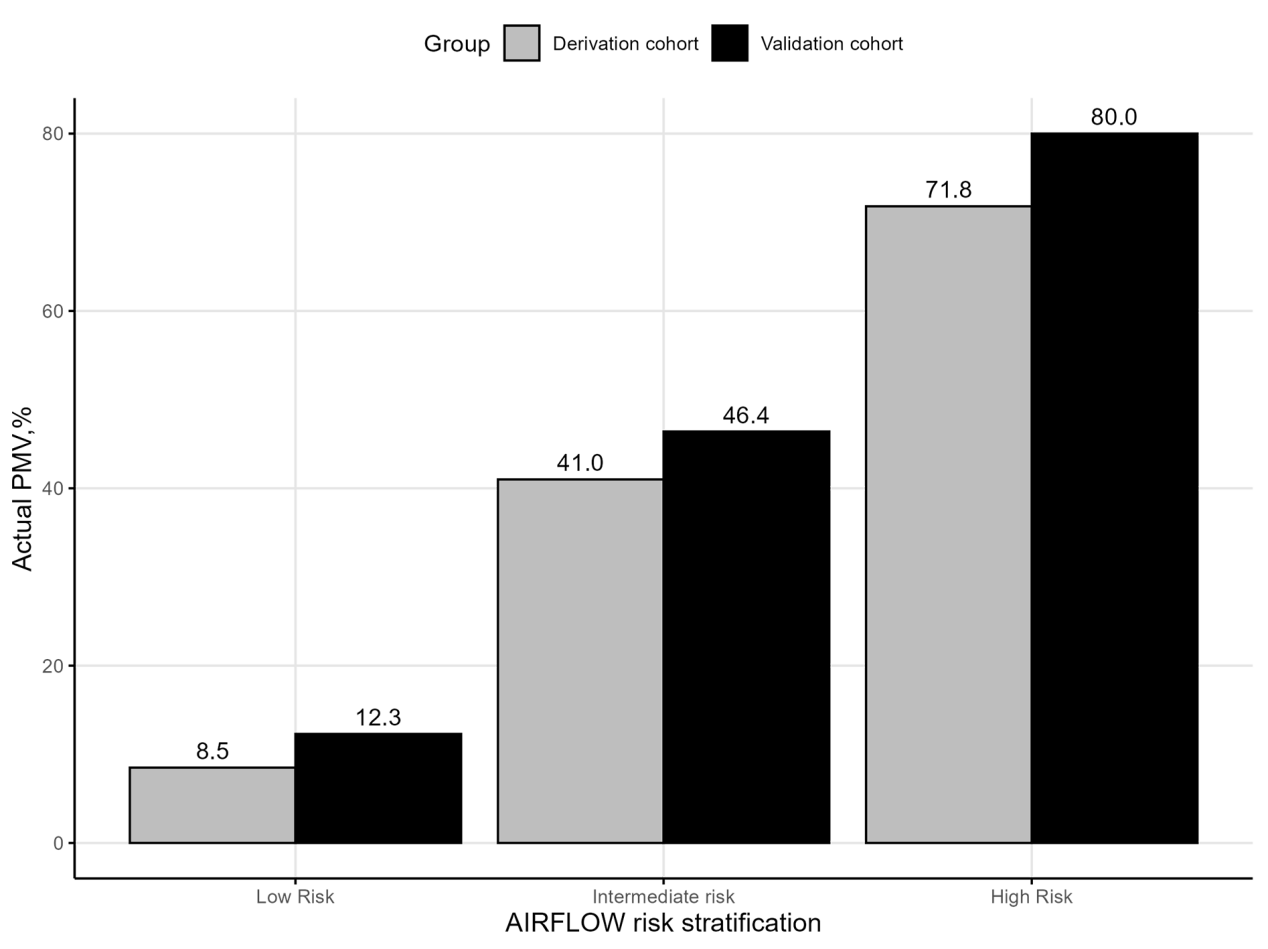


**Figure S3** The association between the three risk stratifications of the sum AIRFLOW score and actual PMV in derivation cohort (A) and in validation cohort (B). PMV prolonged mechanical ventilation.


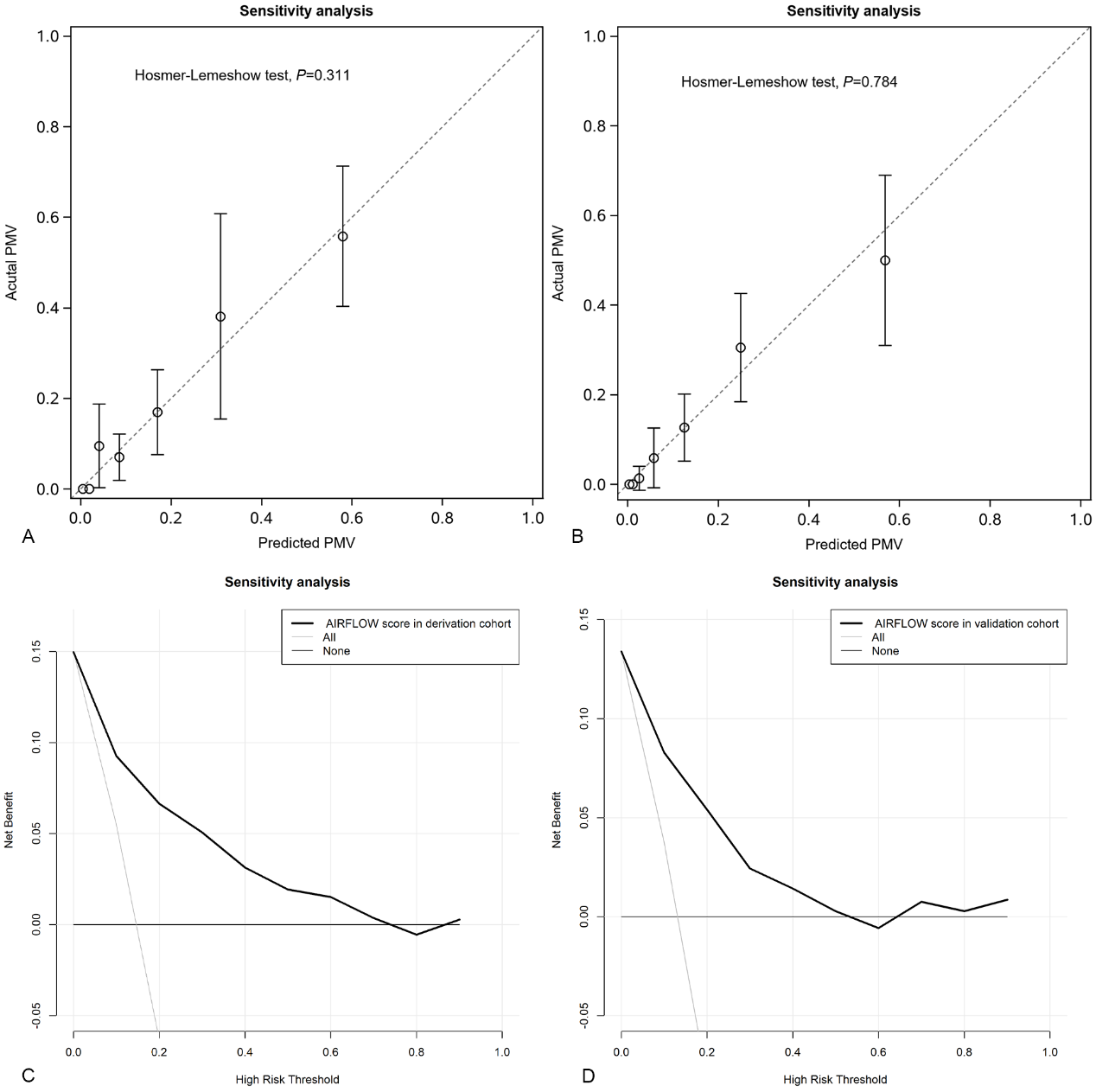


**Figure S4** Sensitivity analysis: calibration plots to assess model in derivation cohort (A) and validation cohort (B). Sensitivity analysis: decision curve analysis demonstrating the net benefit associated with the use of the AIRFLOW score for predicting PMV in derivation cohort (C) and validation cohort (D). PMV prolonged mechanical ventilation


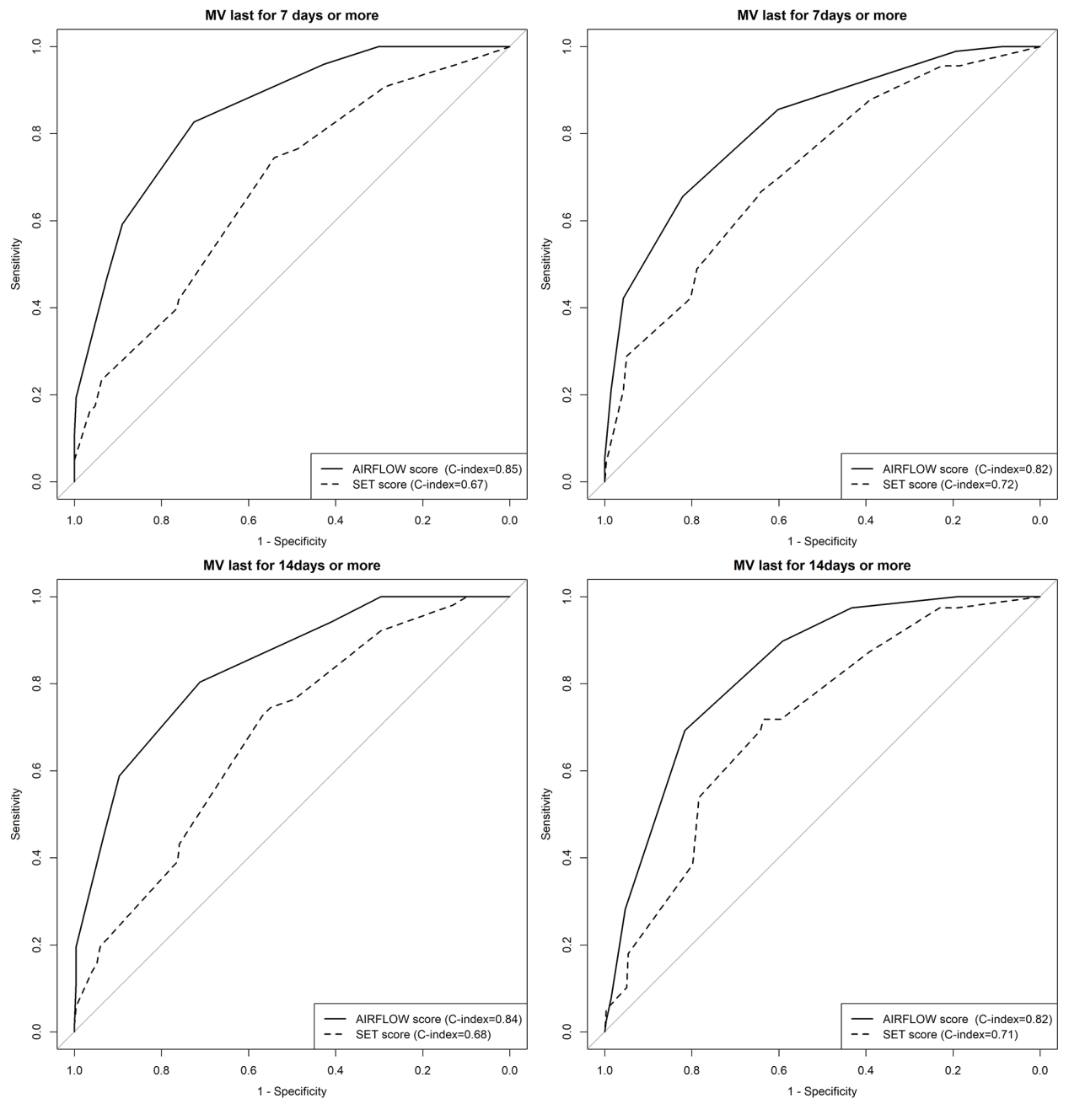


**Figure S5** The performance of the AIRFLOW score compare to the SET score in derivation cohort (A) and validation cohort (B), both with complete data; and in sensitivity analysis for derivation cohort (C) and validation cohort(D), both with complete data. MV mechanical ventilation.

**Table S1** Predicted PMV rate according to sum AIRFLOW score in two cohorts

|  | Derivation Cohort | | Validation Cohort | |
| --- | --- | --- | --- | --- |
| **AIRFLOW sum score** | Number of patients | Predicted  PMV rate | Number of patients | Predicted  PMV rate |
| **0** | 4 | 0.003 | 8 | 0.007 |
| **1** | 19 | 0.006 | 20 | 0.014 |
| **2** | 9 | 0.014 | 33 | 0.031 |
| **3** | 61 | 0.032 | 83 | 0.064 |
| **4** | 47 | 0.073 | 57 | 0.129 |
| **5** | 108 | 0.156 | 91 | 0.244 |
| **6** | 73 | 0.302 | 69 | 0.412 |
| **7** | 27 | 0.499 | 28 | 0.603 |
| **8** | 46 | 0.704 | 16 | 0.767 |
| **9** | 11 | 0.848 | 4 | 0.877 |
| **10** | 12 | 0.929 | 5 | 0.939 |
| **11** | 1 | 0.969 | 0 | - |
| **12** | 1 | 0.986 | 0 | - |

PMV prolonged mechanical ventilation

**Table S2** Components of the SET score grading scale

| **Area of assessment** | **Items** | **Point** |
| --- | --- | --- |
| Neurological function | Dysphagia | 4 |
|  | Observed aspiration | 3 |
|  | GCS on admission<10 | 3 |
| Neurological lesion | Brainstem | 4 |
|  | Space-occupying cerebellar | 3 |
|  | Ischemic infarct >2/3 MCA territory | 4 |
|  | ICH volume>25 ml | 4 |
|  | Diffuse lesion | 3 |
|  | Hydrocephalus | 4 |
| General organ function/procedures | (Neuro) surgical intervention | 2 |
|  | Additional respiratory disease | 3 |
|  | PaO_2_/FiO_2_ <150 | 2 |
|  | APS (of APACHE II) >20 | 4 |
|  | Lung injury score >1 | 2 |
|  | Sepsis | 3 |
| **Total scores：** | | |

Heidelberg NICU in-house scoring tool originally used for estimation of at least 2 weeks of ventilatory support(1)

SET stroke-related early tracheostomy, GCS Glasgow coma scale, MCA middle cerebral artery, ICH intracerebral hemorrhage, PaO2 arterial partial pressure of oxygen, FiO2 fractional inspired oxygen, APS acute physiology score, APACHEII acute physiology and chronic health evaluation II, LIS lung injury score.

The number of patients with complete items for the SET score is 353 in the derivation group, 339 in the validation group, and 304 and 310 in the sensitivity analysis for the derivation and validation groups, respectively.

1. Schönenberger S, Al-Suwaidan F, et al. The SETscore to Predict Tracheostomy Need in Cerebrovascular Neurocritical Care Patients. Neurocrit Care. (2016) 25:94-104.
